# Association Between Health Literacy, Electronic Health Literacy, Disease-Specific Knowledge, and Health-Related Quality of Life Among Adults With Chronic Obstructive Pulmonary Disease: Cross-Sectional Study

**DOI:** 10.2196/12165

**Published:** 2019-06-06

**Authors:** Michael Stellefson, Samantha R Paige, Julia M Alber, Beth H Chaney, Don Chaney, Avery Apperson, Arjun Mohan

**Affiliations:** 1 Department of Health Education and Promotion East Carolina University Greenville, NC United States; 2 STEM Translational Communication Center College of Journalism and Communications University of Florida Gainesville, FL United States; 3 Department of Kinesiology and Public Health College of Science & Mathematics California Polytechnic State University San Luis Obispo, CA United States; 4 Division of Pulmonary, Critical Care and Sleep Medicine Department of Internal Medicine East Carolina University Brody School of Medicine Greenville, NC United States

**Keywords:** COPD, eHealth, health-related quality of life, health literacy, patient education, health status, internet

## Abstract

**Background:**

Despite the relatively high prevalence of low health literacy among individuals living with chronic obstructive pulmonary disease (COPD), limited empirical attention has been paid to the cognitive and health literacy–related skills that can uniquely influence patients’ health-related quality of life (HRQoL) outcomes.

**Objective:**

The aim of this study was to examine how health literacy, electronic health (eHealth) literacy, and COPD knowledge are associated with both generic and lung-specific HRQoL in people living with COPD.

**Methods:**

Adults from the COPD Foundation’s National Research Registry (n=174) completed a cross-sectional Web-based survey that assessed sociodemographic characteristics, comorbidity status, COPD knowledge, health literacy, eHealth literacy, and generic/lung-specific HRQoL. Hierarchical linear regression models were tested to examine the roles of health literacy and eHealth literacy on generic (model 1) and lung-specific (model 2) HRQoL, after accounting for socioeconomic and comorbidity covariates. Spearman rank correlations examined associations between ordinal HRQoL items and statistically significant hierarchical predictor variables.

**Results:**

After adjusting for confounding factors, health literacy, eHealth literacy, and COPD knowledge accounted for an additional 9% of variance in generic HRQoL (total adjusted *R*^2^=21%; *F*_9,164_=6.09, *P*<.001). Health literacy (*b*=.08, SE 0.02, 95% CI 0.04-0.12) was the only predictor positively associated with generic HRQoL (*P*<.001). Adding health literacy, eHealth literacy, and COPD knowledge as predictors explained an additional 7.40% of variance in lung-specific HRQoL (total adjusted *R*^2^=26.4%; *F*_8,161_=8.59, *P*<.001). Following adjustment for covariates, both health literacy (*b*=2.63, SE 0.84, 95% CI 0.96-4.29, *P*<.001) and eHealth literacy (*b*=1.41, SE 0.67, 95% CI 0.09-2.73, *P*<.001) were positively associated with lung-specific HRQoL. Health literacy was positively associated with most lung-specific HRQoL indicators (ie, cough frequency, chest tightness, activity limitation at home, confidence leaving home, sleep quality, and energy level), whereas eHealth literacy was positively associated with 5 of 8 (60%) lung-specific HRQoL indicators. Upon controlling for confounders, COPD knowledge (*b*=−.56, SE 0.29, 95% CI −1.22 to −0.004, *P*<.05) was inversely associated with lung-specific HRQoL.

**Conclusions:**

Health literacy, but not eHealth literacy, was positively associated with generic HRQoL. However, both health literacy and eHealth literacy were positively associated with lung-specific HRQoL, with higher COPD knowledge indicative of lower lung-specific HRQoL. These results confirm the importance of considering health and eHealth literacy levels when designing patient education programs for people living with COPD. Future research should explore the impact of delivering interventions aimed at improving eHealth and health literacy among patients with COPD, particularly when disease self-management goals are to enhance HRQoL.

## Introduction

### Background

Chronic obstructive pulmonary disease (COPD) is a leading cause of death in the United States, affecting up to 15 million US adults [1,2]. An estimated 65 million people across the globe are living with moderate to very severe COPD [3] contributing to significant morbidity and mortality [4]. The most significant symptoms in patients with severe COPD include shortness of breath (dyspnea), anxiety, depression, and sleep disturbances [5]. Nearly 1 million patients with COPD are hospitalized annually for acute disease exacerbations, with 20% of hospitalized patients readmitted within 30 days of being discharged [6-8]. Readmission is often because of low levels of hospital discharge readiness and confusion regarding discharge instructions [9]. Compared with other chronic diseases such as heart disease and diabetes, treatment adherence in COPD is lower [10], with only about half of medication doses taken as prescribed to treat dyspnea [11]. Many patients with COPD as well as their families have difficulty understanding medication directions and self-management guidelines, which inhibits them from experiencing the health benefits of current medical treatments. People living with COPD report low knowledge in several self-management domains [12], which leads to uncertainty about how to live with their condition [13]. Consequently, patients with COPD describe feeling frustrated, trapped, and socially isolated by their disease [14].

Living with COPD can significantly affect physical, mental, and emotional domains of health-related quality of life (HRQoL) [15]; however, the impact of COPD on HRQoL varies across age categories and presence of comorbidities. For example, among patients living with COPD, age tends to be inversely related to HRQoL, with younger patients faring worse on various HRQoL domains [15]. Similar levels of dyspnea produce greater influence on HRQoL among middle-aged adults as compared with older adults living with COPD. People living with COPD also experience significant burden as a result of comorbidities [16,17], which appear to contribute to poor clinical outcomes [18,16]. A history of previous breathing exacerbations represents another major determinant of lung-specific HRQoL among patients with stable COPD [16]. Surprisingly, only about one-third of patients create an action plan with their provider(s) on how to address potential breathing exacerbations [12].

Patients living with COPD generally receive little guidance in terms of how to recognize and avoid breathing exacerbations. Most patients can only answer about two-thirds of questions about their condition correctly [19], and 16% do not even know what an exacerbation is [20]. Inadequate access to disease-specific education on self-management is a major problem in COPD [21], which hinders the patients’ ability to manage symptoms and utilize health care effectively. Areas of knowledge deficiency include inadequate understanding of lung functioning and lack of information on lifestyle factors affected by COPD. Patients who are younger, attend pulmonary rehabilitation, and have more time since their COPD diagnosis have higher knowledge than older patients without access to pulmonary rehabilitation [21]. Nevertheless, patients who report attendance at pulmonary rehabilitation are only able to answer a few more knowledge questions correctly, on average, as compared with patients not enrolled in pulmonary rehabilitation [21].

Low health literacy, or reduced capacity to understand, evaluate, and act on health information, is associated with worse self-management, more severe COPD, learned helplessness, and lower respiratory-specific HRQoL [22,23]. Puente-Maestue and colleagues [24] found that over 50% of COPD patients have low health literacy, which is often compounded by cognitive impairments resulting from hypoxemia as well as by secondary comorbidities, such as depression and anxiety, which influence learning, comprehension, and decision making [23]. COPD patients with inadequate health literacy visit the emergency room and hospital more often, report greater difficulties carrying out their daily living activities, are more dependent on others, and experience higher rates of multimorbidity [24,25]. Low HRQoL places significant health and economic burdens on these patients as well as the health care system. Furthermore, prior research indicates low health literacy levels and lack of disease-specific knowledge also contribute to elevated emergency room and hospital visits [22], whereas higher levels of eHealth literacy result in greater patient knowledge of their diagnosis and better self-management behaviors [26]. In addition, regardless of lung functioning, patients with low health literacy scored worse on patient-reported outcomes. Limited health literacy can also compromise patient-provider communication about medication management, which interferes with a patient’s ability to acquire and use prescribed medications [24,25,27,28].

Although there are multiple communication modes and channels for COPD patient education, many patients are left to primarily locate answers to their disease-related questions outside of primary practice settings through self-directed sources such as the internet and Web-based support groups [26,29]. A growing number of patients are beginning to access social media and mobile health apps for disease management support [30]. For example, 1 study found that almost 80% of patients living with COPD maintain an active social media account [20]. Accordingly, pharmaceutical companies, medical equipment companies, and hospital systems now sponsor numerous online communities and social media websites dedicated to COPD management. Popular social media websites, such as YouTube, demonstrate some potential to positively reach and engage patients living with COPD [31]; however, educational content and quality on social media varies [32]. Moreover, despite the increased availability of Web-based health information on COPD, patients still experience difficulties accessing evidence-based health information about their condition on the internet. Many patients diagnosed with COPD lack confidence in their ability to distinguish between high- and low-quality sources of Web-based health information [26]. Measuring eHealth literacy, or the ability to find, understand, evaluate, and apply health information from the internet to address health problems, is becoming increasingly important as patients with COPD continue to seek health education and medical advice from various internet-based sources.

### Purpose

Although lower health literacy is associated with poorer HRQoL in patients with COPD, we do not yet know which aspects of HRQoL may be most impacted by inadequate health literacy skills. Despite the relatively high prevalence of low health literacy among individuals with COPD, limited empirical attention has been paid to the cognitive and health literacy skills that can uniquely influence patients’ general health status (generic HRQoL) and health outcomes faced primarily by people living with COPD (lung-specific HRQoL). Therefore, it remains unclear whether distinct types of health literacy are associated with both generic and lung-specific HRQoL among adults living with COPD. In addition, very few studies have examined the role that eHealth literacy may play in affecting HRQoL in people living with COPD [26]. The potential moderating role of COPD knowledge on relationships between health/eHealth literacy and HRQoL is also yet to be explored. It is expected that higher COPD knowledge could significantly increase (or enhance) the effects of health literacy and eHealth literacy on lung-specific HRQoL. The purpose of this study was to assess how health literacy, eHealth literacy, and COPD-related knowledge are associated with both generic and lung-specific HRQoL in people living with COPD, particularly after accounting for factors known to be associated with HRQoL, such as socioeconomic status and living with a comorbidity. The following 4 hypotheses were tested during this study:

1. Hypothesis 1: Health literacy, eHealth literacy, and COPD knowledge will be positively associated with generic HRQoL among patients living with COPD, even after controlling for potentially confounding factors affecting HRQoL.
2. Hypothesis 2: Health literacy, eHealth literacy, and COPD knowledge will be positively associated with lung-specific HRQoL among patients living with COPD, even after controlling for potentially confounding factors affecting HRQoL.
3. Hypothesis 3: COPD knowledge will moderate the effect of health literacy on lung-specific HRQoL, such that higher COPD knowledge will significantly increase (or enhance) the effect of health literacy on lung-specific HRQoL.
4. Hypothesis 4: COPD knowledge will moderate the effect of eHealth literacy on lung-specific HRQoL, such that higher COPD knowledge will significantly increase (or enhance) the effect of health literacy on lung-specific HRQoL.

## Methods

### Sample

Adults from the COPD Foundation’s National Research Registry were recruited to participate. This confidential database includes individuals from all 50 states of the United States who self-report physician or specialist-diagnosed COPD. Enrolled individuals can opt-in to being contacted about potential research opportunities that are promoted for patients enrolled in the Registry. Patients who choose to opt-in complete informed consent documentation indicating that they agree to be contacted about potential patient-oriented research opportunities. Enrolled adults older than 40 years with a valid email address were eligible to participate in this study (N=1270). COPD develops most often in adults older than 40 years [33]. Over three-quarters of eligible patients were older than 60 years (981/1270, 77.2%), and over half were female (703/1270, 55.35%). Most eligible patients were white (1184/1270, 93.23%). Patients who self-reported being unable to speak English were excluded because of lack of access to linguistically-appropriate instrumentation for all measures.

### Procedures

Eligible participants received an email invitation to participate in a Web-based survey powered by Qualtrics. All eligible participants were required to provide electronic informed consent before completing the survey. Institutional Review Board (IRB) approval for the study was obtained from both a hospital-based regulatory body overseeing studies of Registry members, as well as a university-sponsored IRB. Participants were notified that their email address would be entered a random drawing to receive a small electronic gift card for their participation. Due to restrictions stipulating that registrants only be contacted once about opportunities to participate in research, the investigators were unable to recontact nonresponders (n=1071).

### Measures

Demographic questions assessed several sociodemographic characteristics, including age (years), sex, race (white, black or African-American, Asian or Pacific Islander, American Indian or Alaska Native, Mixed Race, or other), marital status (now married, now widowed, never married, divorced, or separated), and education level (less than 8th grade, 8th–11th grade, completed high school, some college, college graduate, or postgraduate). Participants were also asked to affirm (yes/no) if they were currently living with 1 or more comorbidities (diabetes, hypertension, heart disease, cancer, stroke-related symptoms, arthritis or joint problems, or other health problems or conditions). Respondents reporting any of these diseases/disorders were coded as *living with comorbidities*, whereas respondents indicating none of these other conditions were marked as *not living with comorbidities*.

COPD knowledge was measured using the Chronic Obstructive Pulmonary Disease Knowledge Questionnaire (COPD-Q) [34]. The COPD-Q is a valid and reliable 13-item self-administered true/false questionnaire assessing COPD knowledge in patients, regardless of their health literacy. This instrument aids researchers and clinicians in identifying gaps in patients’ knowledge about COPD. COPD-Q scores demonstrate acceptable internal consistency (Cronbach alpha=.72) and are significantly related to health literacy and educational attainment [35]. The Spanish version of the COPD-Q has also demonstrated good internal consistency (Cronbach alpha=.85) and high reliability, with an intraclass correlation efficient of .84 for the total score [36].

The 3-item Health Literacy Screening Questionnaire (HLSQ) [37] was used to measure general health literacy. Use of screening questions to assess general health literacy is effective in detecting inadequate health literacy; these items have also been validated against widely used measures of health literacy [38-40]. HLSQ items assess the degree to which participants possess (1) problems with learning health information, (2) self-efficacy completing health-related forms, and (3) problems reading health information on Likert scales ranging from 1 (problems with health literacy) to 5 (no problems with health literacy). HLSQ scores in this study demonstrated marginal internal consistency (Cronbach alpha=.64).

eHealth literacy was assessed using an 8-item rating scale called the eHealth Literacy Scale (eHEALS) [41]. This brief scale assesses one’s perceived ability to find, understand, and appraise health information from Web-based sources and apply knowledge to address health concerns. eHEALS is a reliable computer-based measure of patients’ knowledge and self-efficacy for obtaining and evaluating Web-based health resources [42]. Responders are asked to indicate their level of agreement with 8 statements describing their Web-based health information–seeking experiences on a Likert scale ranging from 1 (strongly disagree) to 5 (strongly agree). Mean Likert scale responses were computed for the 8-item eHEALS. Data collected using the eHEALS in this study showed high internal consistency (Cronbach alpha=.90).

Generic HRQoL was measured using the EuroQol (EQ)-5D [43], an instrument that assesses the following 5 relevant domains of HRQoL: (1) mobility, (2) self-care, (3) usual activity, (4) pain, and (5) anxiety/depression. In each domain, participants are asked to select from 3 responses ranging from *no problems* (level 1) to *some problems* (level 2) to *inability or extreme difficulty* (level 3). Responses are used to quantify an individual’s unique health state by using a 5-digit descriptor ranging from 11111 for perfect health to 33333 for worst possible health. Weights are applied to score responses on each of the 5 domains and then converted to a single index value ranging from 0 to 1, where a score of 1 represents a perfect health state. This index value is based on a value set derived from a Time Trade Off valuation study reflecting data collected in the US population [44]. For post hoc correlational analyses, item scores on each EuroQol-5D (EQ-5D) dimension were reverse scored to facilitate result interpretability (ie, *no problems* originally marked as level 1 were recoded to *3* to reflect higher self-report on HRQoL dimension). The EQ-5D is self-administered, written at a 7th-grade level and can be completed in 5 to 10 min [44]. Data collected with the EQ-5D in this study showed satisfactory internal consistency (Cronbach alpha=.74).

Lung-specific HRQoL was assessed using the COPD Assessment Test (CAT) [45]. CAT is a widely used 8-item self-reported questionnaire that quantifies the impact of COPD symptoms. Respondents are asked to self-report the extent to which they are affected by coughing, chest congestion, chest tightness, breathlessness, difficulty leaving their home, sleep quality, and low energy levels. Item responses range from 1 (no problems) to 5 (significant difficulties). For result interpretability, responses to all 8 items were reverse scored before computing the total CAT scores for each patient. After reverse scoring CAT items, lower scores indicated greater difficulties on relevant dimensions of lung-specific HRQoL (ie, score of *1* indicated significant difficulties on dimension of lung-specific HRQoL). Total possible CAT scores can range from 0 (mild) to 40 (very severe) [45,46]. Among clinically stable COPD patients, total CAT scores are highly correlated with scores on the St. George’s Respiratory Questionnaire [45], which is another commonly used, yet lengthy (50-item), instrument that measures lung-specific HRQoL in people with obstructive airway disease.

### Data Analysis

Frequency statistics were used to summarize categorical data, whereas means (SDs) were computed to describe interval data. Missing items from multi-item scales were mean-imputed if at least half of the scale items were completed. Correlations between main study variables were analyzed using Pearson *r* correlation coefficients.

Hierarchical linear regressions were computed to examine the roles of health literacy and eHealth literacy on generic (model 1) and lung-specific (model 2) HRQoL by sequentially adding predictors into 2 blocks within each model. To control for the effects of demographic covariates and comorbidity status on the dependent variables of generic and lung-specific HRQoL, sociodemographic factors (ie, age, gender, race, ethnicity, marital status, and education level) and comorbidity (present/not present) were entered in block 1 as potential confounding factors affecting each type of HRQoL. Because we were primarily interested in the additional effects of COPD-related knowledge, health literacy, and eHealth literacy above and beyond these covariates, predictor variables were subsequently entered in block 2 of each model. To determine whether health literacy, eHealth literacy, and COPD knowledge provided any significant increment in the amount of variance explained in generic (model 1) and lung-specific HRQoL (model 2), *F*-test statistics were evaluated to determine statistically significant *R*^2^ changes in explained variance (%) at each step of the analysis. Unstandardized beta coefficients were examined for each control/predictor variable, and interaction effects were assessed by interpreting multiple *R*^2^ and adjusted *R*^2^*.* Tolerance and variance inflation factor (VIF) estimates for each hierarchical model were computed to ensure that tolerance estimates were below 0.10 and VIF were less than 10 [47]. In addition, Cohen *f*^2^ is a useful measure of local effect size appropriate for hierarchical regression models [48], and this was calculated for each hierarchical linear regression in this study. According to guidelines by Cohen [49], *f*^2^≥0.02, *f*^2^≥0.15, and *f*^2^≥0.35 represent small, medium, and large effect sizes, respectively.

Associations between ordinally scaled HRQoL items and statistically significant predictor variables from hierarchical analyses were assessed using Spearman rank correlation procedures. In the third (and final) step of the hierarchical regression analyses, interaction terms of eHealth literacy×COPD knowledge and health literacy*COPD knowledge were entered in both models, one predicting generic HRQoL and another predicting lung-specific HRQoL. All statistical analyses were conducted using IBM SPSS Statistics version 24 (SPSS Inc, Chicago, IL, USA), with values of *P*<.05 considered statistically significant.

## Results

### Respondent Characteristics

Overall, 199 adults with COPD submitted responses to the Web-based survey. List-wise deletion was used to remove cases that contained missing data (n=25); therefore, responses from 174 of 1270 patients (response rate=13.70%) were analyzed. Table 1 presents overall characteristics of the study sample (n=174). Participants reported a mean age of 66.06 years (SD 9.43). Slightly over half of the sample were female (88/174, 50.6%), and the clear majority were white (168/174, 96.6%). The sample was also well-educated, with over 80% (147/174, 84.5%) of the sample reporting at least some college education. Most participants were married (106/174, 60.9%); however, almost one-quarter were divorced or separated (42/174, 24.1%).

**Table 1:** Overall characteristics of the study sample (N=174).

Variable		Study participants
Age (years), mean (SD)		66.06 (9.43)
**Sex, n (%)**		
	Female	88 (50.6)
	Male	86 (49.4)
**Race, n (%)**		
	White	168 (96.6)
	Black	3 (1.7)
	Asian or Pacific Islander	1 (0.6)
	American Indian or Alaska Native	1 (0.6)
	Mixed race	1 (0.6)
**Ethnicity, n (%)**		
	Hispanic	4 (2.3)
	Non-Hispanic	170 (97.7)
**Education level, n (%)**		
	8th to 11th grade	5 (2.9)
	High school graduate	22 (12.6)
	Some college	83 (47.7)
	College graduate	37 (21.3)
	Postgraduate degree	27 (15.5)
**Marital status, n (%)**		
	Married	106 (60.9)
	Widowed	16 (9.2)
	Never married	10 (5.7)
	Divorced or separated	42 (24.1)
**Comorbidity, n (%)**		
	Yes	143 (82.2)
	No	31 (17.8)

### Health Literacy, Electronic Health Literacy, and Chronic Obstructive Pulmonary Disease Knowledge

The mean score on HLSQ items measuring health literacy was 4.52 (SD 0.62), which was higher than self-reported scores on the 8-item eHEALS measure of eHealth literacy (mean 3.63, SD 0.71). On average, respondents answered almost 10 (mean 9.81, SD 1.63) out of 13 questions correctly on the COPD-Q measuring knowledge related to COPD.

### Health Status Measures

The mean EQ-5D score was 0.723 (SD 0.18) on the EQ-5D index scale measuring generic HRQoL from 0 to 1, whereas the mean score on the CAT measuring lung-specific HRQoL on a scale of 0 to 40 was 24.02 (SD 6.65), indicating moderate lung-specific HRQoL. EQ-5D index scores were positively associated with CAT scores (*r*=.61, *P*<.001). More than 80% of the sample (143/174, 82.2%) reported living with at least one comorbidity.

### Hypothesis Testing

#### Test of Hypothesis 1: Health Literacy, Electronic Health Literacy, and Chronic Obstructive Pulmonary Disease Knowledge on Generic Health-Related Quality of Life

In the hierarchical regression model examining effects of health literacy, eHealth literacy, and COPD knowledge on generic HRQoL, demographic characteristics and comorbidity (step 1) accounted for 16.0% (adjusted *R*^2^=13%) of the variance in generic HRQoL, *F*_6,167_=5.30, *P*<.001. Table 2 shows that older age (*b*=.01, SE 0.001, *P*<.01) and higher education level (*b*=.04, SE 0.13, *P*<.01) were associated with better generic HRQoL. Introducing health literacy, eHealth literacy, and COPD knowledge as predictor variables (step 2) explained an additional 9% of variance in generic HRQoL (adjusted *R*^2^=21%), *F*_9,164_=6.09, *P*<.001. After adjusting for confounding factors, health literacy (*b*=.08, SE 0.02, 95% CI 0.04-0.12) was the only predictor variable positively associated with generic HRQoL (*P*<.001). Cohen *f *^2^ for the effects of health literacy, eHealth literacy, and COPD knowledge on generic HRQOL was 0.12, which is a medium effect size.

**Table 2:** Hierarchical linear regression model examining effects of health literacy, electronic health literacy, and chronic obstructive pulmonary disease knowledge on generic health-related quality of life (centered at the mean).

Predictor		*R*^2^ change	*b*	SE *b*	95% CI
**Step 1**		.16^a^			
	Age^b^	—^c^	.01^d^	0.001	0.002 to 0.01
	Gender (0=male, 1=female)	—	.02	0.03	−0.04 to 0.07
	Race (0=nonwhite, 1=white)	—	−.01	0.02	−0.05 to 0.04
	Marital status (0=unmarried, 1=married)	—	−.01	0.01	−0.03 to 0.01
	Education (1=less than 8 years, 6=postgraduate)	—	.04^d^	0.01	0.02 to 0.07
	Comorbidity (0=no, 1=yes)	—	−.06	0.03	−0.13 to 0.004
**Step 2**		.09^a^			
	COPD^e^ knowledge^a^	—	−.01	0.01	−0.03 to 0.002
	Health literacy^a^	—	.08^a^	0.02	0.04 to 0.12
	Electronic health literacy^a^	—	.02	0.02	−0.01 to 0.06
Total *R*^2^		.25^a^	—	—	—
Adjusted *R*^2^		.21^a^	—	—	—

^a^*P*<.001.

^b^Centered at the mean.

^c^Not applicable.

^d^*P*<.01.

^e^COPD: chronic obstructive pulmonary disease.

**Table 3:** Spearman rank correlations between EuroQoL-5D dimension scores and statistically significant independent variables emerging from hierarchical linear regression model predicting generic health-related quality of life.

EuroQol-5D dimensions	Age	Education level	Comorbidity (yes=1 or no=0)^a^	Health literacy	Electronic health literacy
(1) Mobility	.11 (.16)	.15 (.05)	−.78 (.43)	.20^b^ (.01)	.15^c^ (.049)
(2) Self-care	.12 (.13)	.18^c^ (.02)	−1.66 (.10)	.42 (<.001)	.23^b^ (.003)
(3) Usual activities	.10 (.20)	.22^b^ (.004)	−.60 (.55)	.23^b^ (.003)	.19^c^ (.01)
(4) Pain/discomfort	.25^b^ (.001)	.25^b^ (.001)	−2.37^c^ (.02)	.29 (<.001)	.05 (.49)
(5) Anxiety/depression	.33^d^ (<.001)	.12 (.11)	−.46 (.64)	.22^b^ (.004)	.11 (.16)

^a^Results of Mann-Whitney Wilcoxon test.

^b^*P*<.01.

^c^*P*<.05.

Table 3 lists correlations between EQ-5D dimension scores and statistically significant predictor variables emerging from the 2-stage hierarchical linear regression model predicting generic HRQoL. Age, education, and health literacy were all significantly (*P*<.05) associated with at least one EQ-5D dimension score. Older participants reported less difficulty managing pain/discomfort (ρ=.25, *P*=.01) and anxiety/depression (ρ=.33, *P*<.001). However, age was not significantly associated with mobility (ρ=.11, *P*=.16), self-care ability (ρ=.12, *P*=.13), or engagement in usual daily activities (ρ=.10, *P*=.20). Higher educational attainment was associated with better ability to wash or dress oneself (ρ=.18, *P*=.05), greater engagement in usual activities such as housework and family or leisure time (ρ=.22, *P*=.004), and less difficulty with pain and discomfort (ρ=.25, *P*=.001). Education level was not significantly associated with mobility or anxiety/depression, although the association between education and mobility approached statistical significance (ρ=.15, *P*=.05). Participants living without comorbidities reported significantly less pain and discomfort (*U*=−2.37, *P*=.02, *η*^2^=0.03) but no significant associations with any other dimension of generic HRQoL. Higher health literacy scores were associated with significantly (*P*<.05) better scores on all 5 EQ-5D dimensions: mobility (ρ=.20, *P*=.01); self-care (ρ=.42, *P*<.001); usual activities (ρ=.23, *P*=.003); pain/discomfort (ρ=.29, *P*<.001); and anxiety/discomfort (ρ=.22, *P*=.004). eHealth literacy was positively associated with 3 generic HRQoL dimensions: mobility (ρ=.15, *P*=.049), self-care (ρ=.23, *P*=.003), and usual activities (ρ=.19, *P*=.01).

#### Test of Hypothesis 2: Health Literacy, Electronic Health Literacy, and Chronic Obstructive Pulmonary Disease Knowledge on Lung-Specific Health-Related Quality of Life

In the hierarchical regression model examining effects of health literacy, eHealth literacy, and COPD knowledge on lung-specific HRQoL, demographic characteristics and comorbidity status (step 1) accounted for 22.5% (adjusted *R*^2^=19.7%) of the variance in lung-specific HRQoL, *F*_6,163_=7.90, *P*<.001. Table 4 shows that older age (b=.21, SE 0.05, *P*<.001), higher education level (b=1.87, SE 0.47, *P*<.001), and living with no comorbidities (b=−2.85, SE 1.120, *P*<.05) were associated with better lung-specific HRQoL scores. Adding health literacy, eHealth literacy, and COPD knowledge as predictor variables (step 2) explained an additional 7.4% of variance in lung-specific HRQoL (adjusted *R*^2^=26.4%), *F*_8,161_=8.59, *P*<.001. After adjusting for potentially confounding factors, health literacy (*b*=2.63, SE 0.84, 95% CI 0.96-4.29, *P*<.001) and eHealth literacy (*b*=1.41, SE 0.67, 95% CI 0.09-2.73, *P*<.001) were positively associated with lung-specific HRQoL. Interestingly, after adjusting for confounding variables, COPD knowledge (*b*=−.56, SE 0.29, 95% CI −1.22 to −0.004, *P*<.05) was inversely associated with lung-specific HRQoL. In other words, higher COPD knowledge was related to lower lung-specific HRQoL among people living with COPD. Cohen *f*^2^ for the effects of health literacy, eHealth literacy and COPD knowledge on lung specific HRQoL was 0.13, which is a medium effect size.

Table 5 describes correlations between CAT item scores and 6 statistically significant predictor variables emerging from the 2-stage hierarchical linear regression model. A total of 5 of the 6 predictor variables were significantly (*P*<.05) associated with at least three CAT item scores, except for COPD knowledge, which was not significantly associated with any CAT item. Increased age was associated with better symptom scores related to cough frequency (ρ=.23, *P*=.002), chest congestion (ρ=.21, *P*=.005), chest tightness (ρ=.30, *P*<.001), sleep quality (ρ=.32, *P*<.001), and energy level (ρ=.22, *P*=.003). Older participants also demonstrated greater confidence leaving home (ρ=.18, *P*=.015). Education level was positively associated (*P*<.05) with better scores on every CAT item, except for chest congestion (ρ=.10, *P*=.17). Participants without comorbidities reported less challenges with chest tightness (*U*=−2.46, *P*=.014, *η*^2^=0.03), more confidence leaving home (*U*=−2.20, *P*=.03, *η*^2^=0.03), and better energy levels (*U*=−2.90, *P*=.004, *η*^2^=0.04). Health literacy was positively associated with scores on most lung-specific symptoms, including cough frequency (ρ=.18, *P*=.02), chest tightness (ρ=.25, *P*=.001), activity limitation at home (ρ=.24, *P*=.002), confidence leaving home (ρ=.29, *P*<.001), sleep quality (ρ=.25, *P*=.001), and energy level (ρ=.30, *P*<.001). eHealth literacy scores were also positively associated with over 60% (5/8) of the CAT items: chest congestion (ρ=.19, *P*=.013), chest tightness (ρ=.17, *P*=.02), activity limitations at home (ρ=.19, *P*=.01), sleep quality (ρ=.15, *P*=.04), and energy level (ρ=.18, *P*=.02).

**Table 4:** Hierarchical linear regression model examining effects of health literacy, electronic health literacy, and chronic obstructive pulmonary disease knowledge on lung-specific health-related quality of life (centered at the mean).

Predictor		*R*^2^ change		*b*		SE *b*		95% CI	
**Step 1**		.23^a^		—^b^		—		—	
	Age^c^		—		.21^a^		0.05		0.11 to 0.31
	Gender (0=male, 1=female)		—		.72		0.98		−1.22 to 2.66
	Race (0=nonwhite; 1=white)		—		−.64		0.82		−2.25 to 0.97
	Marital status (0=unmarried, 1=married)		—		−.21		0.37		−0.95 to 0.53
	Education (1=less than 8 years, 6=postgraduate)		—		1.87^a^		0.47		0.94 to 2.80
	Comorbidity (0=no, 1=yes)		—		−2.85^e^		1.12		−5.21 to –0.49
**Step 2**		.09^a^		—		—		—	
	COPD^e^ knowledge^c^		—		−.56^c^		0.28		−1.12 to −0.004
	Health literacy^c^		—		2.63^f^		0.84		0.96 to 4.29
	Electronic health literacy		—		1.41^f^		0.67		0.09 to 2.73
Total *R*^2^		.32^a^		—		—		—	
Adjusted *R*^2^		.28^a^		—		—		—	

^a^*P*<.001.

^b^Not applicable.

^c^Centered at the mean.

^d^*P*<.05.

^e^COPD: chronic obstructive pulmonary disease.

^f^*P*<.01.

**Table 5:** Spearman rank correlations between Chronic Obstructive Pulmonary Disease-Assessment Test Item scores and statistically significant predictor variables emerging from hierarchical linear regression models. Values in parentheses indicate *P* values.

CAT^a^ items	Age	Education level	Comorbidity (yes=1/no=0)^b^	COPD^c^ knowledge	Health literacy	Electronic health literacy
(1) Cough frequency	.23^d^ (.002)	.20^d^ (.009)	−1.18 (.24)	−.14 (.07)	.18^e^ (.02)	.15 (.06)
(2) Chest congestion	.21^d^ (.005)	.10 (.17)	−1.40 (.16)	−.05 (.51)	.15 (.06)	.19^d^ (.013)
(3) Chest tightness	.30 (<.001)	.31 (<.001)	−2.46^e^ (.014)	−.06 (.45)	.25^d^ (.001)	.17^e^ (.02)
(4) Breathlessness walking uphill	.10 (.19)	.23^d^ (.002)	−.03 (.98)	.03 (.67)	.12 (.13)	.08 (.29)
(5) Activity limitations at home	.117 (.12)	.35 (<.001)	−1.83 (.07)	−.06 (.46)	.235^d^ (.002)	.19^e^ (.01)
(6) Confidence leaving home	.18^e^ (.02)	.24^d^ (.001)	−2.20^e^(.03)	.04 (.62)	.29 (<.001)	.14 (.06)
(7) Sleep quality	.32 (<.001)	.24^d^ (.001)	−1.71 (.08)	−.01 (.93)	.25^d^ (.001)	.15^e^ (.04)
(8) Energy level	.22^d^ (.003)	.31 (<.001)	−2.90^d^ (.004)	−.01 (.93)	.30 (<.001)	.18^e^ (.02)

^a^CAT: COPD Assessment Test.

^b^Results of Mann-Whitney Wilcoxon test.

^c^COPD: chronic obstructive pulmonary disease.

^d^*P*<.01.

^e^*P*<.05.

**Table 6:** Moderating effect of chronic obstructive pulmonary disease knowledge on the relationship between health literacy and lung-specific health-related quality of life (centered at the mean).

Predictor		*R*^2^ change	*b*	SE *b*	95% CI
**Step 1**		.23^a^	—^b^	—	—
	Age^c^	—	0.21^a^	0.05	0.11 to 0.31
	Gender (0=male, 1=female)	—	0.72	0.98	−1.22 to 2.66
	Race (0=nonwhite, 1=white)	—	−0.64	0.82	−2.25 to 0.97
	Marital status (0=unmarried, 1=married)	—	−0.21	0.37	−0.95 to 0.053
	Education (1=less than 8 years, 6=postgraduate)	—	1.87^a^	0.47	0.94 to 2.80
	Comorbidity (0=no, 1=yes)	—	–2.85^e^	1.20	–5.21 to –0.49
**Step 2**		.07^a^	—	—	—
	COPD^e^ knowledge^c^	—	−0.45	0.28	−1.00 to 0.11
	Health literacy^c^	—	2.63^f^	0.84	0.96 to 4.29
**Step 3**		.02 (*P*=.055)	—	—	—
	COPD knowledge health literacy	—	0.89	0.46	–0.02 to 1.80
Total *R*^2^		.31^a^	—	—	—
Adjusted *R*^2^		.27^a^	—	—	—

^a^*P*<.001.

^b^Not applicable.

^c^Centered at the mean.

^d^*P*<.05.

^e^COPD: chronic obstructive pulmonary disease.

^f^*P*<.01.

#### Test of Hypothesis 3: Moderating Effect of Chronic Obstructive Pulmonary Disease Knowledge on the Relationship Between Health Literacy and Lung-Specific Health-Related Quality of Life

The interaction between COPD knowledge and health literacy (step 3) did not account for significantly more variance in lung-specific HRQoL than COPD knowledge and health literacy alone, *R*^2^ change=.01, *P*=.10 (Table 6). Moreover, the interaction effect of health literacy and lung-specific HRQoL (*b*=.890, SE 0.461) only approached statistical significance (*P*=.055). Greater knowledge of COPD did not significantly increase (or enhance) the effect of health literacy on lung-specific HRQoL in this sample of patients living with COPD. Cohen *f*^2^ for the moderating effects of COPD knowledge on health literacy and lung-specific HRQoL was 0.12, which is a medium effect size.

#### Test of Hypothesis 4: Moderating Effect of Chronic Obstructive Pulmonary Disease Knowledge on the Relationship Between Electronic Health Literacy and Lung-Specific Health-Related Quality of Life

The interaction between COPD knowledge and eHealth literacy (step 3) also did not account for significantly more variance in lung-specific HRQoL than COPD knowledge and health literacy did alone, *R*^2^ change=.01, *P*=.28 (Table 7). Greater knowledge of COPD did not significantly increase (or enhance) the effect of eHealth literacy on lung-specific HRQoL in the sample of patients living with COPD. Cohen *f*^2^ for the moderating effects of COPD knowledge on eHealth literacy and lung-specific HRQoL was 0.07, which is a small effect size.

**Table 7:** Moderating effect of chronic obstructive pulmonary disease knowledge on the relationship between electronic health literacy and lung-specific health-related quality of life.

Predictor		*R*^2^ change	*b*	SE *b*	95% CI
**Step 1**		.23^a^	—^b^	—	—
	Age^c^	—	0.21^a^	0.05	0.11 to 0.31
	Gender (0=male, 1=female).	—	0.72	0.98	−1.22 to 2.66
	Race (0=nonwhite; 1=white)	—	−0.64	0.82	−2.25 to 0.97
	Marital status (0=unmarried, 1=married)	—	−0.21	0.37	−0.95 to 0.53
	Education (1=less than 8 years, 6=postgraduate)	—	1.87^a^	0.47	0.94 to 2.80
	Comorbidity (0=no, 1=yes)	—	−2.85^d^	1.20	−5.21 to –0.49
**Step 2**		.05^e^	—	—	—
	COPD^f^ knowledge^c^	—	−0.56	0.29	−1.13 to 0.02
	Electronic health literacy^c^	—	1.99^e^	0.66	0.69 to 3.29
**Step 3**		.002	—	—	—
	COPD knowledge electronic health literacy	—	−.025	0.40	−1.04 to 0.54
Total *R*^2^		.28^a^	—	—	—
Adjusted *R*^2^		.24^a^	—	—	—

^a^*P*<.001.

^b^Not applicable.

^c^Centered at the mean.

^d^*P*<.05.

^e^*P*<.01.

^f^COPD: chronic obstructive pulmonary disease.

## Discussion

### Principal Findings

This study aimed to assess how health literacy, COPD-related knowledge, and eHealth literacy are associated with both generic HRQoL and lung-specific HRQoL for people diagnosed with COPD while controlling for the effect of comorbid conditions, socioeconomic status, education level, and other factors known to be associated with HRQoL. Overall, results provide support for the importance of considering both health and eHealth literacy levels when developing patient education and self-management support programs for people living with COPD. More specifically, results revealed that health literacy was significantly associated with all 5 EQ-5D dimensions measuring generic HRQoL, whereas eHealth literacy was associated with significantly better scores on 3 generic HRQoL dimensions (mobility, self-care, and usual activities). Both health and eHealth literacies were positively associated with lung-specific HRQoL; however, higher levels of COPD knowledge did not significantly enhance the impact of health literacy or eHealth literacy on lung-specific HRQoL. These results also signified approximately moderate (*f*^2^≥0.15) effect sizes according to Cohen’s guidelines [49], specifically with the effects of health literacy and eHealth literacy on both generic and lung-specific HRQoL yielding effect size coefficients in the moderate/medium range. Results indicate the need to consider both eHealth literacy and health literacy levels of patients when developing patient education and support programs, particularly when the intended outcome is to enhance lung-specific quality of life. In addition, given that COPD knowledge did not demonstrate an impact on health literacy and eHealth literacy, future research should explore addressing these 2 constructs in the context of patient self-management programs rather than traditional patient education programs that solely focus on knowledge dissemination alone.

### Influence of Health Literacy, Electronic Health Literacy, and Chronic Obstructive Pulmonary Disease Knowledge on Generic Health-Related Quality of Life

Overall, results showed similar HRQoL mean scores and correlates (ie, age, education level) to previous studies. HRQoL is generally lower for individuals living with COPD compared with the general population [50]. This study found that the mean EQ-5D score for measuring generic HRQoL among participants with COPD was .723 (SD 0.18) on a 0 to 1 scale, with the higher score meaning better health. In a report of nationally representative values for the US adult population, the average EQ-5D score for both males and females is reported to be above 0.80 for most age brackets, except for adults 80 years and older, which is slightly lower, but above the mean EQ-5D score for participants in this study [51]. The significant associations with age and education level to at least one dimension as assessed here by the EQ-5D are consistent with previous studies that have demonstrated that age and education influence HRQoL [52-54]. According to van Manen and colleagues [54], HRQoL is a multidimensional construct that encompasses both a physical and mental component. Age negatively affects general health and physical function, whereas it positively influences mental health; and in general, those with higher, formal education levels report better mental health [54]. In addition, education level has been found to be a significant predictor of poor health outcomes with COPD [55] while directly affecting health status through associations with patient ability to navigate a complex health care system [56,57].

However, as this study showed, education and age were not significantly associated with all dimensions of HRQoL, as measured by the EQ-5D; therefore, more research should be conducted to better explain which dimensions are influenced by these variables and in what ways. Furthermore, after adjusting for confounding variables, including age and education level, only health literacy was found to be significantly associated with generic HRQoL. These findings are consistent with 2 previous research studies [22,24] that indicate limited health literacy influences general HRQoL of people living with COPD. Specifically, these studies indicate that lower health literacy levels are associated with worse COPD severity and greater levels of helplessness reported among patients living with COPD [22,24]. In addition, these research results suggest that the role of health literacy in COPD may be more important than education when it comes to health outcomes [22].

Although the current investigation demonstrates a strong association between health literacy and general HRQoL, even when controlling for sociodemographic variables such as education and age, eHealth literacy and COPD knowledge were not statistically associated with all 5 EQ-5D dimensions. However, eHealth literacy was significantly associated with scores on 3 generic HRQoL dimensions, including mobility, usual activities, and self-care. Collectively, these dimensions reflect the ability of a patient to manage their illness, and prior research supports that higher levels of eHealth literacy can improve patient self-management of COPD [26]. Nevertheless, there are few existing studies examining associations between eHealth literacy with self-reported health status, which limits what inferences can be made. More longitudinal studies are needed to examine whether better self-management and HRQoL outcomes are experienced by patients with COPD who are more eHealth literate [58].

The results regarding COPD knowledge support previous research findings that the transfer of disease-specific knowledge does not necessarily have a long-term impact on behavior change that could lead to improved quality of life for patients living with COPD [59]. In fact, these findings imply that COPD knowledge alone is not enough to improve HRQoL; however, the potential for using COPD knowledge in conjunction with skill acquisition approaches and self-efficacy–building strategies, with a goal of reaching behavior change, could prove to be collective core elements that are helpful in achieving long-term impact on general HRQoL of patients with COPD [59]. More research is warranted to assess the specific role COPD knowledge plays in factors impacting behavior changes that are associated with general HRQoL.

### Influence of Health Literacy, Electronic Health Literacy, and Chronic Obstructive Pulmonary Disease Knowledge on Lung-Specific Health-Related Quality of Life

The results, overall, showed that after adjusting for potentially confounding variables, health literacy and eHealth literacy levels were significantly associated with lung-specific HRQoL. The impact of distressing COPD symptoms, such as dyspnea, fatigue, coughing, anxiety, wheezing, pain interference, and breathlessness, can help explain much of the variance in lung-specific HRQoL among patients living with the disease [60-62]. This study sought to evaluate the impact of health literacy, eHealth literacy, and COPD knowledge on lung-specific HRQoL. These findings support previous research on the associations between poor health literacy and COPD-related outcomes, revealing lower levels of health literacy relate to worse COPD severity, higher levels of COPD helplessness, and poorer levels of lung-specific HRQoL [22]. As such, this study demonstrates similar results to that of past research indicating poor health literacy is a risk factor associated with poor health outcomes for patients living with COPD, including lung-specific HRQoL, and these patients could be more at-risk for COPD-related emergency health care utilization and overall economic cost burdens associated with the disease. Certainly, these results, as they stand, do not provide reason to support causal relationships between health literacy and lower health outcomes, including lung-specific HRQoL; however, Omachi and colleagues [22] suggest that such relationships could exist, and more research on causality is needed. In addition, the results support the need to consider addressing both health literacy and eHealth literacy in COPD patient education programming.

eHealth literacy is also positively associated with patients’ lung-specific HRQoL in this sample. The eHealth literacy scores are comparable with those in other studies assessing eHealth literacy in the general older adult population [63], as a majority of adults self-reported moderate to above average levels of eHealth literacy. Patients living with COPD who can evaluate the quality of Web-based health information for use in their own care are more amenable to engaging in formal disease self-management programs and more skilled in making decisions related to their specific diagnosis [64]. Therefore, individuals with COPD who have these skills to delineate the quality of resources found on the internet for clinical decision making to manage the distressing symptoms of COPD have enhanced lung-specific HRQoL. Future patient-centered research should emphasize the importance of patients having better eHealth literacy skills to not only enhance lung-specific HRQoL but also potentially better health outcomes among patients living with COPD.

Although eHealth literacy was positively associated with lung-specific HRQoL, it was not associated with generic HRQoL. Patients with COPD who use Web-based media for health-related purposes readily disclose unmet needs related to respiratory symptoms, including cough, mucus production, and dyspnea [65]. Rather than accessing generic health information, patients who go on the internet may be more inclined to access health information that is relevant to COPD and its symptomology [29]. This includes self-management topics focused more on lung-specific outcomes (eg, shortness of breath and chest congestion) rather than generic HRQoL outcomes (eg, mobility, pain, or discomfort). Therefore, high eHealth literate patients with COPD may be accessing self-management information about behavior changes that have the potential to improve lung functioning yet not directly lead to enhanced general HRQoL. More research is needed to understand eHealth approaches to self-management education for individuals with COPD, and how eHealth skill-building strategies impact overall, functional health outcomes for patients.

### Effect of Chronic Obstructive Pulmonary Disease Knowledge on the Relationship Between Health Literacy, Electronic Health Literacy, and Generic/Lung-Specific Health-Related Quality of Life

Greater COPD knowledge did not enhance the effect of health literacy or eHealth literacy on general or lung-specific HRQoL. COPD knowledge, however, was inversely related to lung-specific HRQoL; therefore, the higher the knowledge level, the lower was the self-reported lung-specific HRQoL of study participants. This finding was not anticipated, but one explanation for this result is that even with increased knowledge of the disease, patients might lack the skills or self-efficacy to change their behaviors that are linked to one’s disease state, impacting their lung-specific HRQoL. In addition, it is possible that more knowledge related to the serious diagnosis may result in higher levels of anxiety, fear, and even depression, which could, in turn, negatively affect their general and/or lung-specific HRQoL [66].

### Limitations

This study has several limitations related to recruitment techniques employed, a low sample size, and lack of a representative sample. Despite collaborating with the COPD Foundation’s National Research Registry, this Web-based survey had a low response rate of patients with COPD (13.7%) as compared with other internet surveys conducted with the general population [67]. Our recruitment approach was limited to a single email. Given that recruitment approaches are rarely effective at reaching target enrollment with a one-time invitation, our 13.7% response rate is quite commendable for this patient population. Patients with COPD are generally older and experience frequent hospitalizations that prevent them from engaging in their everyday routines, which may include email checking. This study recruited from a research registry, which requires patients to register for program, and may not be representative of all patients with COPD and may have contributed to the low response rate. This selection bias is essentially unavoidable when using research registries but should be considered when interpreting results. In addition, the magnitude of this bias toward the main results is unknown, as data on unenrolled patients from the underlying population base are unavailable for further analyses. It would only help internal validity to have data on patients not enrolled in the national registry on the exact factors being assessed in this study. Therefore, generalizability of results may be affected and limited to patients in similar registries. Future studies should explore if results from this study are similar with recruitment that includes both Web-based and non-Web-based techniques.

The statistically significant associations found in this cross-sectional study cannot be used to establish cause-and-effect relationships; however, use of hierarchical predictor variable entry provided some advantages when attempting to identify relationships between health/eHealth literacy, COPD knowledge, and relevant health-related outcomes. The construct of eHealth literacy was measured with eHEALS, a widely used scale that has been questioned in the age of new media where communication technologies such as social media are pervasive [68,69]. Studies assessing population eHealth literacy may consider using more updated measures now available to researchers [68,70].

In addition, low socioeconomic status minorities living with COPD were underrepresented. Most participants in this study identified as being white and highly educated, which is not reflective of the sociodemographics of COPD [71]. As mentioned elsewhere [26], future studies assessing health information technology use among patients with COPD should seek to recruit larger numbers of participants with more diverse racial or ethnic, geographic, and economic characteristics. Recruitment should also entail more inclusive strategies than email only, which will likely encourage participation among patients being treated in community-based and clinical settings.

### Conclusions

Although previous research has shown a relatively high prevalence of low health literacy among individuals living with COPD, little empirical attention has been directed at exploring the cognitive and health literacy–related skills that can influence patients’ HRQoL. Findings from this study indicated that health literacy, but not eHealth literacy, was positively associated with generic HRQoL. In addition, health literacy and eHealth literacy were found to be positively associated with lung-specific HRQoL, with higher COPD knowledge associated with lower lung-specific HRQoL. These results confirm the importance of considering patient health and eHealth literacy levels when designing self-management support programs for people living with COPD. Future research should explore the impact of delivering COPD interventions aimed at improving eHealth and health literacy, particularly when self-management program goals include improving HRQoL outcomes.

## Abbreviations

CAT: COPD Assessment Test
COPD: chronic obstructive pulmonary disease
COPD-Q: Chronic Obstructive Pulmonary Disease Knowledge Questionnaire
eHealth: electronic health
eHEALS: eHealth Literacy Scale
EQ-5D: EuroQol-5D
HLSQ: Health Literacy Screening Questionnaire
HRQoL: health-related quality of life
IRB: institutional review board
VIF: variance inflation factor
